# Oral Health in Pregnant Chinese Women in Singapore: A Call to Go beyond the Traditional Clinical Care

**DOI:** 10.3390/healthcare6030077

**Published:** 2018-07-09

**Authors:** Preethi Balan, Hong-Gu He, Fengchunzhi Cao, Mun Loke Wong, Yap-Seng Chong, Violeta Lopez, Shu-E. Soh, Chaminda Jayampath Seneviratne

**Affiliations:** 1 Discipline of Oral Sciences, Faculty of Dentistry, National University of Singapore, Singapore 119083, Singapore; p.preethidr@gmail.com (P.B.); denwml@nus.edu.sg (M.L.W.); 2 Alice Lee Centre for Nursing Studies, Yong Loo Lin School of Medicine, National University of Singapore, Singapore 117597, Singapore; nurhhg@nus.edu.sg (H.G.H.); caofengchunzhi@gmail.com (F.C.); nurvl@nus.edu.sg (V.L.); 3 Singapore Institute for Clinical Sciences, Agency for Science, Technology and Research, Singapore 117549, Singapore; obgcys@nus.edu.sg (Y.-S.C.); paesse@nus.edu.sg (S.-E.S.); 4 Department of Obstetrics and Gynaecology, Yong Loo Lin School of Medicine, National University of Singapore, Singapore 119074, Singapore; 5 Department of Paediatrics, Yong Loo Lin School of Medicine, National University of Singapore, Singapore 119228, Singapore

**Keywords:** attitudes, Chinese, knowledge, oral health, practice, pregnant women

## Abstract

**Objective**: To examine the correlations among oral health knowledge, attitude, practices and oral disease among pregnant Chinese women in Singapore. **Methods**: A descriptive correlational study was conducted in pregnant Chinese women in Singapore. A questionnaire was used to collect data of oral health knowledge, attitude and practices. Plaque index scores were used to assess the oral health of subjects. **Results**: A total of 82 pregnant women participated in the study, out of whom 38% showed adequate oral health knowledge, nearly half of them achieved adequate and oral health attitude and practice scores while 34% had good Plaque index scores. The lower income group had higher experience of self-reported dental problems during pregnancy than those in the higher income group (*p* = 0.03). There were significant positive correlations between scores of oral health practice, attitude and oral health knowledge levels. The plaque index scores negatively correlated with the oral health practice scores (*p* = 0.02). **Conclusions**: Our findings provided evidence that oral health knowledge, attitude and practices among Chinese pregnant women were not optimal which implies the importance of promoting their oral health during pregnancy through the improvement of knowledge and attitudes. This would facilitate formulation and implementation of appropriate oral health promotion policies.

## 1. Introduction

Pregnancy brings about progressive physical and psychological changes in women along with hormonal changes. The increased physical and emotional demands during pregnancy might contribute to the neglect in oral hygiene leading to poor oral health. Inadequate oral hygiene and hormonal changes increase the risk of developing oral health problems such as gingivitis and periodontitis [1]. Gingivitis is the most commonly reported oral problem during pregnancy, with a prevalence of 60 to 75% [2]. Women with pre-existing gingivitis may also show significant exacerbation of the condition during pregnancy [3]. In the first trimester, women commonly experience morning sickness, which leads to increased acidity in the oral cavity and this high acidity erodes the tooth enamel [4]. In the second trimester, pregnancy granuloma has been commonly documented, which frequently occurs in areas of inflamed gingivitis and other recurrent irritation areas. A systematic review has concluded that oral disease especially periodontitis is a contributing factor in pre-term low birth weight babies due to the elevation of inflammatory markers and transmission of oral bacteria into the feto-placental unit [5]. Therefore, a critical step in preventing oral diseases and its complications during pregnancy is to practice good oral hygiene habits.

The literature suggests that the common factors for poor oral health during pregnancy include low oral health literacy [6,7], stress in pregnancy [8], high cost of dental services [9,10], failure of prioritizing oral care [11,12] and poor social economic status [13]. Despite good tooth brushing habits, usage of adjuvant dental products such as dental floss, interdental brush, and mouth rinse is low [14,15]. Further, with the generally lower education levels, this group of women has a poor understanding of the information gathered which ultimately contributes to poor oral health practices [16].

Singapore with its unique blend of ethnicities and traditions brings a different perspective to knowledge on oral health, child bearing practices and confinement period where general health practices may be modified [16]. This is especially significant in Chinese population, where dietary habits incorporate the “hot and cold” elements [17]. These cultural beliefs and practices can at times facilitate or act as barriers to oral health maintenance and access to health care services. Hence, in this study, we have taken oral health knowledge, attitude, practices and plaque index scores as oral health determinants to gain insights into the oral health of pregnant Chinese women in Singapore. A better understanding of oral health as influenced by knowledge, attitudes and practices will be useful in formulating appropriate oral health promotion interventions.

The aims of the study were to investigate various oral health determinants (oral health knowledge, attitude, practices and plaque index scores) in pregnant Chinese women in Singapore and their correlations. Moreover, the association of oral health status during pregnancy with the oral health determinants and the social demographic factors was also explored.

## 2. Materials and Methods

### 2.1. Study Design, Setting and Participants

This study adopted a cross-sectional, descriptive and correlational research design to investigate the current oral health related knowledge, attitudes and practices in Singapore Chinese pregnant women. Participants were recruited from the Obstetrics clinic of National University Hospital, a public tertiary hospital in Singapore, from October 2015 to April 2016. The study recruited 82 women who met the following selection criteria:

Inclusion criteria: (a) Singaporean or permanent residents of Chinese ethnicity; (b) Aged 21–50 years; and (c) Pregnant at less than 24 weeks’ gestation.

Exclusion criteria: Women with any systemic health conditions such as diabetes, cardiovascular diseases or blood dyscrasias were excluded from the study to ensure there was no influence of these factors on the oral health.

The sample size was calculated through power analysis for Pearson product-moment correlation coefficient. As there were no similar studies examining the correlation among the three dependent variables, we anticipated a conventional medium effect size of 0.25, and hence a minimum of 82 participants were needed to achieve a 80% power at 0.05 (two-sided) significance level [18].

### 2.2. Outcomes and Measurements

A structured self-administered questionnaire was used to explore the demographic background, oral health knowledge, attitude and practices of the participants. The questionnaire was self-developed based on the Pregnancy Risk Assessment Monitoring System (PRAMS) [19], Rustvold Oral Health Knowledge Inventory (ROHKI), and Oral Health Attitudes Questionnaire (OHAQ) [19,20] and experts’ opinion. The questionnaire consisted of five parts. Part one included socio-demographic characteristics including age, number of pregnancies, monthly household income, highest educational level attained, and smoking history. In Part two, oral health knowledge was assessed with questions on diet and the etiology of oral disease, safety of dental treatment and use of dental X-rays during pregnancy, association of poor oral health and adverse pregnancy outcomes and need for care regimes when oral diseases develop during pregnancy. Part three consisted of oral health attitude questions and participants were asked about their opinions about dental visits and importance of oral health during pregnancy and general health. A five-point Likert scale with responses ranging from 1 (Strongly Disagree) to 5 (Strongly Agree) was used to assess each item in the oral health knowledge and attitude sections. The knowledge on association between adverse pregnancy outcome and maternal dental health was assessed with a three point Likert scale with “Agree”, “Unsure” and “Disagree” as the responses. Part four assessed their oral health practices with closed-ended questions on tooth brushing techniques, frequency of brushing and dental visits. Self-rated oral health status was assessed on a five-point rating scale with responses ranging from 1 (very poor) to 5 (very good). In Part five, the participants were asked if they were experiencing any oral health problems during pregnancy with responses as “yes” or “no” and if they did, details of the problem were noted.

The plaque index was graded by a single trained examiner on a scale from 1 to 3, where 1 = “One third or less than one third of all teeth present have dental plaque/calculus deposits”, 2 = “More than one third of the teeth but less than half of all teeth present have dental plaque/calculus deposits” and 3 = “Half or more than half of all teeth present have dental plaque/calculus”.

Oral health knowledge, attitude and practice and plaque index scores, were considered as the determinants of oral health. The oral health status of participants during pregnancy was determined by self-reported presence of oral problems and was considered the dependent variable.

An overall score was generated for oral health knowledge, attitude and practices by summing the response codes from the questionnaire items. The cut-off for scores of oral health determinant variables were set at the median. Placing the cut-off at the median is a commonly used method to set the cut-off score in health knowledge tests [21]. Accordingly, participants scoring more than the median score were identified as having good performance while participants scoring less than or equal to the median score were considered to have poor performance with respect to oral health attitude, knowledge and practice. The reverse relation was considered for the plaque index score.

### 2.3. Data Collection Procedure

The researchers recruited Chinese women who were currently undergoing prenatal care in the study clinic. Potential participants were given an information sheet explaining the study’s aims, procedures, their responsibility, possible risks and side effects, possible benefits, alternatives to participation, confidentiality of data and voluntary participation. Those who were willing to participate, provided signed consent to take part in the study. Following this, they were given a self-administered questionnaire, which took about 20–30 min to complete. Thereafter, Plaque Index Score was evaluated by examining all the teeth. This was conducted in a private room to ensure privacy. A token of appreciation was given to each participant after they had completed the questionnaire and dental examination.

### 2.4. Ethical Considerations

The study was approved by the Institutional Review Board of the study hospital (NHG DSRB Ref: 2014/00979). Signed informed consent was obtained from all participants upon recruitment. Voluntary participation and confidentiality of the data collection were ensured. Participants could choose to terminate participation at any time.

### 2.5. Statistical Analysis

Data were entered into SPSS^®^ version 22 (IBM SPSS Statistics for Windows, Version 22.0. IBM Corp, Armonk, NY, USA) and analysed. Descriptive statistics were computed to compile data for the demographics and other key variables of interest. As the data were not normally distributed, non-parametric tests were used. Prior to analysis, some of the knowledge and attitude questions were reverse-coded to facilitate an appropriate scoring system for oral health knowledge and attitudes. The responses for income level and educational level were collapsed to form a three levelled response for data analysis. In addition, response for self-perception of oral health status was also categorised for data analysis. As there were many tied ranks in the sample, Kendall’s tau-b (τb) was used to evaluate the statistical significance and correlations between oral health determinants. The association of oral health determinant and sociodemographic factors on the oral health status during pregnancy was analyzed using chi-square or Fisher’s exact. The level of statistical significance was set at *p* ≤ 0.05.

## 3. Results

### 3.1. Socio-Demographics and Self-Reported Clinical Characteristics of Participants

A total of 82 pregnant Chinese women attending their antenatal care in a public tertiary hospital were recruited. The age of the participants ranged from 21 to 43 years (Mean ± SD = 31.8 ± 4.5 years). The majority of participants (98.8%) were married and their mean gestational age was 12.1 weeks (SD = 5.2). The educational levels of the participants ranged from primary school education to Doctor of Philosophy with a preponderance of participants with secondary school/general equivalency diploma (46.3%) and bachelor’s degree (41.4%). All participants were reported to be non-smokers. The majority of the participants (81.7%) intended to go through post-partum confinement practice. Out of the 82 participants, 24.4% reported dental problems since the start of pregnancy. Of the participants who had dental problems during pregnancy, 75% reported bleeding gums. However, only 6.7% visited dentist for treatment of bleeding gums, even though 73.2% of them perceived their oral health status as moderate or poor. More than half of the participants (56.1%) were unsure that there could be a potential association between maternal dental problems and risk of having adverse pregnancy outcomes (Table 1).

### 3.2. Levels of Oral Health Determinants among Participants

**Figure 1:**
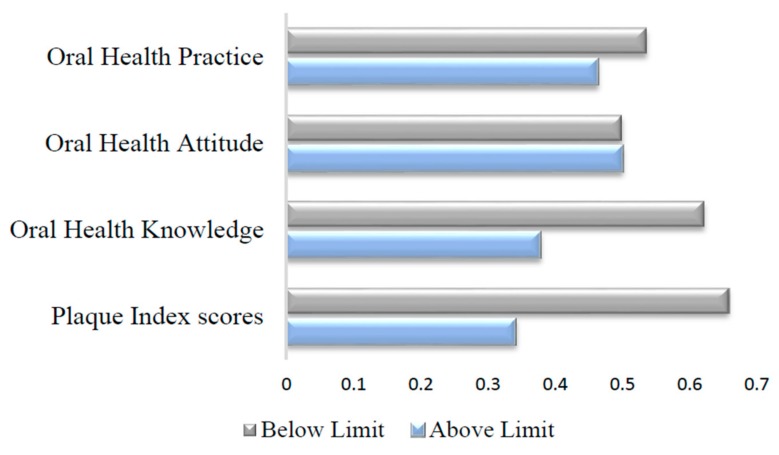
Distribution of scores of oral health determinant factors based on cut-off value.

Table 2 shows the mean scores for oral health knowledge, attitude and practices and dental plaque. The cut-off for oral health determinant’s score was set at the median [21]. Accordingly, 38% of the participants displayed adequate oral health knowledge at cut-off 28. Nearly half of the participants had satisfactory oral health attitude (50%) and practice (46%) scores as defined by the cut-off scores of 28 and 4 respectively. Plaque index scores were good in 34% of participants at cut-off 1 (Figure 1).

### 3.3. Association of Oral Health Status during Pregnancy with the Oral Health Determinant Factors and the Social Demographic Factors

Oral health knowledge scores were higher in respondents not experiencing any dental problems during pregnancy than those who had problems (Table 3). Similarly, the oral health attitude and practice scores were also higher in participants in whom dental problems were absent, although the results were not statistically significant. Furthermore, the plaque index scores were better in participants without dental problems than those who had problems.

Participants who reported having dental problems were older than those who did not report having any dental problems. There was an income-related gradient in the dental problem experience among the respondents. Those who were in the lower income group had a statistically higher experience of dental problems during pregnancy than those in the higher income groups (*p* = 0.03). A similar trend in relation to dental problems was also reported with the educational level, as non-graduate participants appeared to be at higher risk of having dental problems during pregnancy than the more highly educated participants. However, the difference was not statistically significant. This information proves instrumental in identifying the target population which needs auxiliary dental care during pregnancy.

### 3.4. Correlations among Oral Health Determinants

Kendall tau b test provided support for the correlations between the scores for oral health determinants. The plaque index scores negatively correlated with the oral health practice scores (r = −0.2, *p* = 0.02). The scores for oral health practice were shown to be significantly and positively correlated with the levels of oral health attitude (r = 0.2, *p* = 0.01). Furthermore, oral health attitude correlated positively with the oral health knowledge (r = 0.3, *p* < 0.001) (Table 4).

## 4. Discussion

To our knowledge, this is the first study which has explored the oral health behavior and outcomes in the pregnant population in Singapore. Pregnant Chinese women were the focus of this study as the vast majority of Singapore’s population is made up of Chinese (74.3%) [22]. The mean age of the mothers in this study was 31.8 year (SD ± 4.5), which was consistent with the fertility age of Singaporean women (30–34 years) [23]. The majority of the participants (81.7%) intended to go through postpartum confinement practice.

This study indicated bleeding gums as the major dental complaint experienced during pregnancy in 75% of the participants who reported having dental problems. This is consistent with the report on the increase in the prevalence and severity of gingival inflammation occurring during pregnancy [2]. These gingival changes could be the result of hormonal fluctuations or alteration in immune responses during pregnancy [24]. There is an exacerbated inflammatory response to dental plaques leading to swollen gingivae which tend to bleed on provocation. Management of pregnancy gingivitis involves regular dental visits for professional cleaning and monitoring. In addition, educating women about the etiology and prevention of the condition is also essential [25]. However, in this study only 6.7% of the participants with bleeding gums visited the dentist for treatment. This figure is meagre as compared to other countries like Australia, where Thomas et al. reported that 30% of the women accessed dental care during pregnancy [26] and in the US, where 22.7–34% of the pregnant women utilized dental services during pregnancy [27]. A possible consequence of such oral hygiene neglect could be an increased risk for adverse pregnancy outcomes such as pre-term deliveries, low birthweight babies, and preeclampsia, especially in the light of mounting evidence of association between the oral health and birth outcomes [28]. The periodontal health status has been associated with adverse pregnancy outcomes, especially in pregnant women older than 40 years of age [29]. In this study, more than half of the respondents were unsure of such associations while approximately 6% disagreed with this. However, numerous studies have also indicated that most women were unaware of the association between poor oral health and the well-being of the fetus [7,10,30]. This indicates that there is an imperative need to educate pregnant women regarding maintaining their oral health during pregnancy.

Oral health knowledge regarding safety of dental treatment or X-ray examination during pregnancy, causes or presentation of dental caries, care for bleeding gums experienced during pregnancy was found to be poor amongst the participants in the study. Poor knowledge about oral health among pregnant women could be attributed to the prevalence of misconceptions that poor oral health is normal and accepted during pregnancy or that dental treatment can be harmful to the fetus [31]. Confusion over safety of dental treatment has been found to be the most important factor limiting access to dental care among mothers in Greece [32]. Moreover, most pregnant women do not receive enough information about oral health and the importance of dental care prior to and during pregnancy [11,33]. The common consensus is that prevention, diagnosis and treatment of dental conditions including use of dental X-rays (with shielding of abdomen and thyroid) is safe during pregnancy [34]. Dental health professionals must update their knowledge on pregnancy related conditions and their proper management without imposing any adverse impact on the patient and fetus [35]. Referral and consultation to patient’s gynecologist’s or physician should be considered, whenever required.

The oral health attitudes were also observed to be low with half of the participants falling below the cut-off value for desirable attitude. This could perhaps be due to the fact that the most of the women did not consider it important to have a dental check-up during pregnancy and chose to defer it until after pregnancy [33,36]. These misperceptions and erroneous beliefs about oral health care may contribute to the low utilization rate of dental services [37]. This was evident in this study where only 6.7% of participants sought treatment after developing bleeding gums during pregnancy.

**Figure 2:**
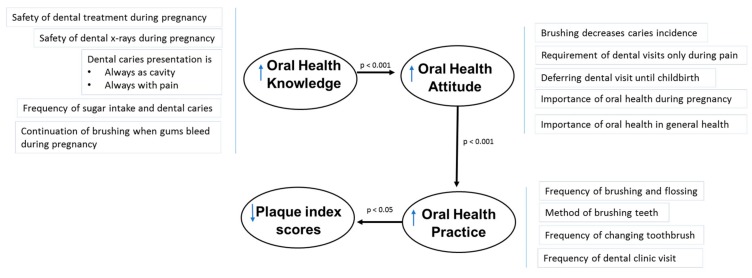
Correlation between the oral health determinant factors.

This study also showed low practice levels in more than half of the participants (54%). Moreover, the plaque index scores, which is an indicator of oral hygiene and a proxy for gingival health [38], was also high in about 66% of participants, suggesting a low level of oral hygiene among the pregnant women. This finding was in line with results from a study by Bressane et al., 2011; reporting inadequate oral health behaviors during pregnancy [39]. Figure 2 summarizes the correlation between these oral health determinant factors. This study also suggested that, while 73.2% of the participants perceived their oral health status to be poor or moderate, they did not consider oral health care as an urgent need and preferred delaying the dental visit until after delivery.

In this study, the oral health status during pregnancy (with or without dental problem) was associated with oral health-determining factors and sociodemographic factors. Although the results were not significant, it was found that the oral health knowledge, attitude and practice scores were lower in the participants who were experiencing oral health problems during pregnancy as compared to those who did not. Similarly, the plaque scores were also higher in participants with dental problems during pregnancy. As the inflammatory response to dental plaque is increased during pregnancy, it is crucial that women take additional effort in oral care to avoid having dental problems during gestation [25]. Oral health, especially the prevention of gingivitis and accumulation of plaque, is very much dependent on the adherence to oral hygiene behaviors and regular dental visits [40]. Pregnancy being a stressful period, with the increased physical and emotional demands, could potentially contribute to stress-related neglect on oral hygiene as was shown by Amin and ElSalhy [30], where almost half of the pregnant women surveyed had instances of forgetting to brush their teeth during pregnancy. The findings also showed that people of low socioeconomic status were significantly more likely to have dental problems during pregnancy than those of higher socioeconomic status (*p* = 0.03).The high cost of dental services is a strong deterrent for dental visits [9,10]. Women with higher household income have the financial capability for regular dental visits and are more likely to seek dental treatments, both preventive and curative, as compared to women with lesser household income [10]. Oral health services are often cited as one of the more expensive services. For example, in a national survey of 801 pregnant women conducted by Cigna Corporation a, only half of the women were observed to have dental insurance. Furthermore, those with dental insurance were twice as likely to visit the dentist [41]. Hence, having dental insurance coverage has been identified as an enabling factor that promotes dental visits [15,30] to potentially bridge the gap.

To add to the aforementioned information, our findings also showed significant positive correlations between oral health knowledge and attitude (r = 0.3), and oral health attitude and practice (r = 0.2). This is consistent with previous findings showing similar correlational strengths [42]. Given that poor oral hygiene practices can result in build-up of dental plaque, oral health practices had significant negative correlations with plaque index scores (r = −0.210). These findings concur with findings from existing literature [43].

As this study was a self-reported survey it may have inherent weaknesses and biases. Our cohort included participants with a gestational age of less than 24 weeks. As oral finding tend to increase with progressing gestational age, assessing oral health at a fixed time point would have helped better correlation of time specific oral findings prevalent during pregnancy with the oral health determining factors. Nevertheless, to our knowledge, this is the first empirical study that has examined oral health determining factors in the Chinese population in Singapore. From this study, it appears that oral health knowledge is lacking in pregnant Chinese women in Singapore. This could possibly result in the adoption of negative attitudes and a lack of emphasis on oral health care. The existence of interactions between culture-related factors such as normative beliefs, knowledge, and behaviors with economic and social factors structure one’s health chances and choices [44]. Unless an individual’s adopted system of assumptions is challenged and revolutionized, it is not possible to bring change to their healthcare behavior patterns. All these pregnancy-related oral health problems could be mitigated with good oral hygiene practices, highlights the importance of oral care during pregnancy [45]. In addition, researches have indicated that oral health knowledge and attitudes do influence the oral health practices, such as tooth brushing habits, use of adjunct dental products and frequency of dental visits. This signals an imperative need to impart oral health education to pregnant women, with the greatest need in the lower socio-economic strata.

Pregnancy is among the best time for information seeking and a good opportunity for improving health knowledge, and pregnant women are generally more motivated to make behavior change for the well-being of the baby [46]. Improving health knowledge and practices will require the support of oral healthcare and prenatal care providers. Communication by health care providers should address the myths and misconceptions many women have about oral health during pregnancy and increase their awareness.

## 5. Conclusions

Maternal periodontal disease has been associated with preterm birth, development of preeclampsia, and delivery of a small-for-gestational age infant. Evidence also suggests that infants and young children acquire caries-causing bacteria from their mothers. Given the considerable effect of oral diseases on pregnant women as well as a child’s downstream oral health, the following future recommendations are proposed based on the findings of the study; firstly, conducting continuous oral health education programmes to heighten awareness of the oral–systemic health and clarifying myths regarding the safety of dental treatment during pregnancy; secondly, check-ups and oral healthcare should be encouraged during the perinatal visits by the healthcare professionals; and lastly, the availability of subsidized oral healthcare services for pregnant women who need such assistance. Equipped with more knowledge, women will have more self-efficacy and motivation for self-monitoring of brushing and flossing habits and subsequently better oral health outcomes.

## Figures and Tables

**Table 1:** Sociodemographic and oral health characteristics (N = 82).

Sociodemographic and Oral Health Characteristics	Mean ± SD	N (%)
**Sociodemographic characteristics**		
Age in years	31.8 (± 4.5)	
Gestational age	12.1 (± 5.2)	
Marital status		
Married		84 (98.8%)
Single		1 (1.2%)
Highest educational level		
Secondary school/diploma		38 (46.3%)
Graduate		34 (41.4%)
Masters/PhD		10 (12.2%)
Monthly household income		
Low (≤2999$)		23 (28.1%)
Medium (3000 to 4999$)		19 (23.2%)
High (≥5000$)		40 (48.8%)
Smoking status		
Yes		0 (0.0%)
No		82 (100%)
Intentions of practicing confinement		
Yes		70 (81.7%)
No		15 (18.3%)
**Oral health characteristics**		
Dental problems during pregnancy (Self-reported)		
Yes		20 (24.4%)
No		62 (75.6%)
Self-reported dental complaints		
Tooth decay		2 (2.4%)
Bleeding gums		15 (18.3%)
Gum boil		0 (0.0%)
Tooth-ache		5 (6.1%)
Tooth injury		1 (1.2%)
Bleeding gums within participants with dental problems during pregnancy		
Yes		15 (75.5%)
No		5 (25.5%)
Participants who visited dentist for bleeding gums during pregnancy		
Yes		1 (6.7%)
No		14 (93.9%)
Self-perception of oral health status		
Good		22 (26.8%)
Poor/moderate		60 (73.2%)
Association between adverse pregnancy outcomes and oral health		
Agree		31(37.8%)
Disagree		5 (6.1%)
Unsure		46 (56.1%)

**Table 2:** Levels of oral health determinant factors (N = 82).

Score	Mean ± SD	Median	Minimum	Maximum
Oral health knowledge	27.5 ± 3.2	28	16.00	37.00
Oral health attitude	27.7 ± 2.9	28	22.00	34.00
Oral health practice	4.4 ± 1.8	4	0.00	9.00
Plaque index	1.4 ± 0.6	1	1.00	3.00

**Table 3:** Association of oral health status with oral health determinant factors and social demographic factors (N = 85).

	Oral Health Status during Pregnancy		
	Problem present N = 20 (24.4%)	Problem absent N = 62 (75.6%)	*p*-value
Oral health determinants			
Oral health knowledge score (Mean) ^a^	38.9	42.3	0.572
Oral health attitude score (Mean) ^a^	39.3	42.1	0.640
Oral health practice score (Mean) ^a^	40.0	41.9	0.747
Plaque index (Mean) ^a^	44.4	40.5	0.462
Social demographic factors			
Age (Mean) ^a^	34.3	43.8	0.121
Income status (N, %) ^b^			
High (5000$–>10,000$)	5 (12.5)	35 (87.5)	0.03
Medium (3000$–4999$)	7 (36.8)	12 (63.2)	0.890
Low (<1000$–2999$)	8 (34.8)	15 (65.2)	
Educational level (N, %) ^b^			
Masters/PhD	1 (10.0)	9 (90.0)	0.172
Graduate	7 (20.6)	27 (79.4)	0.291
Secondary school/Diploma	12 (31.6)	26 (68.4)	

^a^ Mann-Whitney test; ^b^ Chi-square test.

**Table 4:** Correlations among oral health determinant factors (N = 82).

	1	2	3	4
1. Oral health knowledge score	1.000			
2. Oral health attitude score	0.300 **	1.000		
3. Oral health practice score	0.056	0.215 *	1.000	
4. Plaque index score	−0.108	−0.081	−0.200 *	1.000

* *p* < 0.05; ** p < 0.01.

## Appendix A

List of abbreviations

SPSS	Statistical Package for the Social Sciences
PRAMS	Pregnancy Risk Assessment Monitoring System
ROHKI	Rustvold Oral Health Knowledge Inventory
OHAQ	Oral Health Attitudes Questionnaire
NHG DSRB	National Healthcare Group Domain Specific Review Boards
NUHS	National University Health System

## References

[1] La Marca-Ghaemmaghami P., Ehlert U. (2015). Stress during Pregnancy. Eur. Psychol..

[2] Silk H., Douglass A.B., Douglass J.M., Silk L. (2008). Oral health during pregnancy. Am. Fam. Phys..

[3] Hey-Hadavi J. (2002). Women’s oral health issues: Sex differences and clinical implications. Women’s Health Prim. Care Clin..

[4] Sherman P.W., Flaxman S.M. (2002). Nausea and vomiting of pregnancy in an evolutionary perspective. Am. J. Obs. Gynecol..

[5] Shanthi V., Vanka A., Bhambal A., Saxena V., Saxena S., Kumar S.S. (2012). Association of pregnant women periodontal status to preterm and low-birth weight babies: A systematic and evidence-based review. Dent. Res. J..

[6] Hom J.M., Lee J.Y., Divaris K., Diane Baker A., Vann W.F. (2012). Oral health literacy and knowledge among patients who are pregnant for the first time. J. Am. Dent. Assoc..

[7] Wu Y.M., Ren F., Chen L.L., Sun W.L., Liu J., Lei L.H., Zhang J., Cao Z. (2014). Possible socioeconomic and oral hygiene behavioural risk factors for self-reported periodontal diseases in women of childbearing age in a Chinese population. Oral Health Prev. Dent..

[8] Le M., Riedy C., Weinstein P., Milgrom P. (2009). Barriers to utilization of dental services during pregnancy: A qualitative analysis. J. Dent. Child..

[9] Buerlein J.K., Horowitz A.M., Child W.L. (2011). Perspectives of Maryland women regarding oral health during pregnancy and early childhood. J. Publ. Health Dent..

[10] George A., Johnson M., Blinkhorn A., Ajwani S., Bhole S., Yeo A.E., Ellis S. (2013). The oral health status, practices and knowledge of pregnant women in south-western Sydney. Aust. Dent. J..

[11] Detman L.A., Cottrell B.H., Denis-Luque M.F. (2010). Exploring dental care misconceptions and barriers in pregnancy. Birth.

[12] Marchi K.S., Fisher-Owen S.A., Weintraub J.A., Yu Z., Braveman P.A. (2010). Most pregnant women in California do not receive dental care: Findings from a population-based study. Publ. Health Rep..

[13] Gilbert G.H. (2005). Racial and socioeconomic disparities in health from population-based research to practice-based research: The example of oral health. J. Dent. Educ..

[14] Hunter L.P., Yount S.M. (2011). Oral health and oral health care practices among low-income pregnant women. J. Midwifery Women’s Health.

[15] Thompson T.A., Cheng D., Strobino D. (2013). Dental cleaning before and during pregnancy among Maryland mothers. Matern. Child Health J..

[16] Chen L.W., Low Y.L., Fok D., Han W.M., Chong Y.S., Gluckman P., Godfrey K., Kwek K., Saw S.M., Soh S.E. (2014). Dietary changes during pregnancy and the postpartum period in Singaporean Chinese, Malay and Indian women: The GUSTO birth cohort study. Public Health Nutr..

[17] Naser E., Mackey S., Arthur D., Klainin-Yobas P., Chen H., Creedy D.K. (2012). An exploratory study of traditional birthing practices of Chinese, Malay and Indian women in Singapore. Midwifery.

[18] Cohen J. (1992). A power primer. Psychol. Bull..

[19] Pregnancy Risk Assessment Monitoring System (PRAMS) Questionnaires.

[20] Rustvold S.R. (2012). Oral Health Knowledge, Attitudes, and Behaviors: Investigation of an Educational Intervention Strategy with at-Risk Females. Doctoral Thesis.

[21] Yuen H.K., Wolf B.J., Bandyopadhyay D., Magruder K.M., Salinas C.F., London S.D. (2009). Oral health knowledge and behavior among adults with diabetes. Diabetes Res. Clin.Pract..

[22] Singapore Department of Statistics (2016). Population Trends.

[23] Singapore Department of Statistics (2016). Population Trends.

[24] Wu M., Chen S.W., Jiang S.Y. (2015). Relationship between gingival inflammation and pregnancy. Med. Inflamm..

[25] Pirie M., Cooke I., Linden G., Irwin C. (2007). Dental manifestations of pregnancy. Obs. Gynaecol..

[26] Thomas N.J., Middleton P.F., Crowther C.A. (2008). Oral and dental health care practices in pregnant women in Australia: A postnatal survey. BMC Pregnancy Childbirth.

[27] Gaffield M.L., Gilbert B.J., Malvitz D.M., Romaguera R. (2001). Oral health during pregnancy: An analysis of information collected by the pregnancy risk assessment monitoring system. J. Am. Dent. Assoc..

[28] Baskaradoss J.K., Geevarghese A., Al Dosari A.A.F. (2012). Causes of Adverse Pregnancy Outcomes and the Role of Maternal Periodontal Status—A Review of the Literature. Open Dent. J..

[29] Capasso F., Vozza I., Capuccio V., Vestri A.R., Polimeni A., Ottolenghi L. (2016). Correlation among periodontal health status, maternal age and pre-term low birth weight. Am. J. Dent..

[30] Amin M., ElSalhy M. (2014). Factors affecting utilization of dental services during pregnancy. J. Periodontol..

[31] George A., Johnson M., Duff M., Ajwani S., Bhole S., Blinkhorn A., Ellis S. (2012). Midwives and oral health care during pregnancy: Perceptions of pregnant women in south-western Sydney, Australia. J. Clin. Nurs..

[32] Hullah E., Turok Y., Nauta M., Yoong W. (2008). Self-reported oral hygiene habits, dental attendance and attitudes to dentistry during pregnancy in a sample of immigrant women in North London. Arch. Gynecol. Obs..

[33] Al Habashneh R., Guthmiller J.M., Levy S., Johnson G.K., Squier C., Dawson D.V., Fang Q. (2005). Factors related to utilization of dental services during pregnancy. J. Clin. Periodontol..

[34] Committee on Health Care for Underserved Women (2013). Oral health care during pregnancy and through the lifespan. Obs. Gynecol..

[35] Naseem M., Khurshid Z., Khan H.A., Niazi F., Zohaib S., Zafar M.S. (2016). Oral health challenges in pregnant women: Recommendations for dental care professionals. Saudi J. Dent. Res..

[36] Chacko V., Shenoy R., Prasy H.E., Agarwal S. (2013). Self-reported awareness of oral health and infant oral health among pregnant women in Mangalore, India—A prenatal survey. Int. J. Health Rehabilit. Sci..

[37] Saddki N., Yusoff A., Hwang Y.L. (2010). Factors associated with dental visit and barriers to utilisation of oral health care services in a sample of antenatal mothers in Hospital Universiti Sains Malaysia. BMC Public Health.

[38] Al-Sufyani G.A., Al-Maweri S.A., Al-Ghashm A.A., Al-Soneidar W.A. (2014). Oral hygiene and gingival health status of children with Down syndrome in Yemen: A cross-sectional study. J. Int. Soc. Prev. Commun. Dent..

[39] Bressane L.B., Costa L.N.B.D.S., Vieira J.M.R., Rebelo M.A.B. (2011). Oral health conditions among pregnant women attended to at a health care center in Manaus, Amazonas, Brazil. Rev. Odonto Cienc..

[40] Deinzer R., Granrath N., Spahl M., Linz S., Waschul B., Herforth A. (2005). Stress, oral health behaviour and clinical outcome. Br. J. Health Psychol..

[41] Cigna Corporation (2015). 2015 Cigna Corporation Healthy Smiles for Mom and Baby: Insights into Expecting and New Mothers’ Oral Health Habits. https://www.cigna.com/assets/docs/newsroom/cigna-study-healthy-smiles-for-mom-and-baby-2015.pdf.

[42] Tang Y., Zhu Y.Q., Wang Y., He Y. (2011). A survey about knowledge, attitude, practice of oral health in pregnant women of one hospital in Shanghai municipality. Shanghai J. Stomatol..

[43] Ifesanya J.U., Ifesanya A.O., Asuzu M.C., Oke G.A. (2010). Determinants of good oral hygiene among pregnant women in Ibadan, south-western Nigeria. Ann. Ib. Postgrad. Med..

[44] Beegle D. (2003). Overcoming the Silence of Generational Poverty: Invisible Literacies. http://www.combarriers.com/TP0151Overcoming.pdf.

[45] Steinberg B.J., Hilton I.V., Iida H., Samelson R. (2013). Oral health and dental care during pregnancy. Dent. Clin. North Am..

[46] Jackson J.T., Quinonez R.B., Kerns A.K., Chuang A., Eidson R.S., Boggess K.A., Weintraub J.A. (2015). Implementing a prenatal oral health program through interprofessional collaboration. J. Dent. Educ..

